# Removal of 4-Ethylphenol and 4-Ethylguaiacol with Polyaniline-Based Compounds in Wine-Like Model Solutions and Red Wine

**DOI:** 10.3390/molecules200814312

**Published:** 2015-08-05

**Authors:** Verónica Carrasco-Sánchez, Amalraj John, Adolfo Marican, Leonardo S. Santos, V. Felipe Laurie

**Affiliations:** 1 School of Agricultural Sciences, Universidad de Talca, Talca 3460000, Chile; E-Mail: vecarrasco@utalca.cl; 2 Laboratory of Asymmetric Synthesis, Chemistry Institute of Natural Resources, Universidad de Talca, Talca 3460000, Chile; E-Mails: johnamalraj@gmail.com (A.J.); amarican@utalca.cl (A.M.); lssantos@utalca.cl (L.S.S.); 3 Nanobiotechnology Division at Universidad de Talca, Fraunhofer Chile Research Foundation—Center for Systems Biotechnology, FCR-CSB, Talca 3460000, Chile

**Keywords:** 4-EP, 4-EG, polyaniline, conjugated polymers, wine, QuEChERS

## Abstract

Volatile phenols, such as 4-ethyphenol (4-EP) and 4-ethylguaiacol (4-EG), are responsible for the “Brett character” found in wines contaminated with Brettanomyces yeast (i.e., barnyard, animal, spicy and smoky aromas). In these trials, we explore the effectiveness of polyaniline-based compounds (polyaniline emeraldin salt (PANI-ES) and polyanaline emeraldin base (PANI-EB)), for the removal of 4-EP and 4-EG from acidic model solutions and red wine. First, a screening study, performed in an acidified 12% ethanol solution, was used to optimize parameters such as contact time and the amount of polymers required to remove 4-EP and 4-EG. Then, the trapping ability of PANI agents towards 4-EP and 4-EG was evaluated in a model solution containing other wine phenolics that could potentially be trapped by PANI (i.e., gallic acid and 4-methylcatechol). The results of this trial showed that both PANI compounds were capable of removing 4-EP, 4-EG, regardless of the presence of other phenolic compounds present at a much higher concentration. Finally, the capturing ability of PANI was evaluated in a red wine sample containing 5 mg·L^−1^ of 4-EP, 5 mg·L^−1^ of 4-EG and 2.03 ± 0.02 g·L^−1^ of total phenolics. The results showed that PANI-EB removed significantly more 4-EP and 4-EG than PANI-ES. For instance, a treatment with 10 mg·mL^−1^ of PANI-EB produced a 67.8% reduction of 4-EP, 50% reduction of 4-EG and 41.38% decrease in total phenols.

## 1. Introduction

Spoilage with Brettanomyces yeast has been widely described as one of the main issues in winemaking due to their potential contribution to off-aromas (i.e., the well-known “Brett” or phenolic character), and the difficulties involved in the elimination of this type of yeast [1].

Brettanomyces is responsible for the transformation of hydroxycinnamic acids (HCAs) into ethylphenols (EPs) by means of two enzymatic steps. First, the hydroxycinnamic acid is decarboxilated into vinylphenol (VPh) by a hydroxycinnamate decarboxilase (HCDC) enzyme, and later reduced to EP by vinylphenol reductase (VPhR) [2,3,4,5]. The above-mentioned reactions may take place during wine elaboration and maturation, particularly during wine aging in wood barrels [6]. Two of the most important compounds produced by Brettanomyces are the volatile phenols 4-ethylphenol and 4-ethylguaiacol. These compounds may range from a few micrograms up to several milligrams per liter, and may affect the wine’s sensory quality depending mainly on its chemical composition (e.g., matrix effects with non-volatile compounds). For instance, it has been reported that when the total concentration of these compounds exceeds 600 µg·L^−1^, the “Brett” character of wines (i.e., barnyard, animal, spicy or smoky aromas) might be undesirable [7]. Instead, lower concentrations of these compounds may positively contribute to the aromatic complexity of wines [7,8,9].

Several remedial approaches have been proposed in order to reduce the concentration of volatile phenols in wines. For instance, the mixing of spoiled with clean wine, the use of reverse osmosis [10], sorption of volatile phenols on yeast lees and cell-walls [11,12,13], molecularly imprinted polymers [14] or esterified cellulose polymers [15]. In this manuscript, we further explore the possibility of using polyaniline-based materials as fining agents for the removal of these compounds from wine.

**Figure 1:**
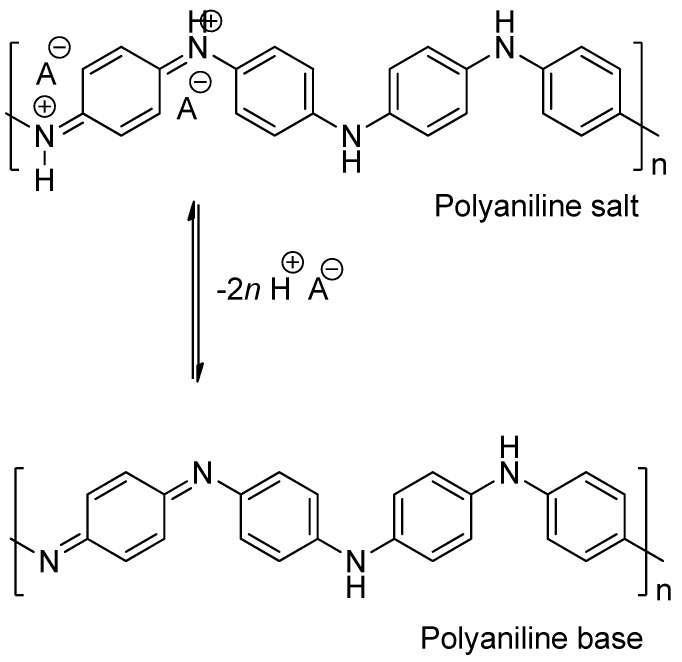
Chemical structure of polyaniline emeraldin salt (PANI-ES) and polyanaline emeraldin base (PANI-EB).

Polyaniline (PANI) materials are a group of compounds which usefulness has been proven in several fields. As indicated elsewhere, the electric conducting capabilities of PANI materials have been used in several applications such as transistors, switches, electrochemical actuators, lightning protection, etc. [16,17]; and their use has also been reported in biomedical applications such as artificial tissue and muscles development [17]. Recently, our research group has proposed the use of these polymers in the emaraldine states (i.e., emeraldine salt, ES and emeraldine base, EB; Figure 1) as potential remediating agents for the removal of unwanted substances from food matrices [18,19]. These polymers are stable at different temperatures and pH levels, are not metabolized by common microorganisms [20], and are relatively inexpensive to produce.

Given the prior results, the aim of this research was to evaluate the effectiveness of the addition of PANI materials for the removal of volatile phenols (i.e., 4-ethylphenol and 4-ethylguaiacol) from wine-like model solutions and wine.

## 2. Results and Discussion

This section includes a series of assays in which polyaniline emeraldin salt (PANI-ES), and polyanaline emeraldin base (PANI-EB) were independently tested as fining agents for the removal of 4-ethylphenol (4-EP) and 4-ethylguaiacol (4-EG) from acidic model solutions and red wine. First, optimization studies were performed in model wine, containing 4-EP and 4EG, to evaluate the polymer dose and contact time required for their removal (Section 2.1). Then, the trapping ability of PANI polymers towards 4-EP and 4-EG was evaluated in a model solution containing other highly concentrated wine phenolics (i.e., gallic acid and 4-methylcatechol) that could potentially be trapped by PANI (Section 2.2). Finally, the capturing ability of PANI was evaluated in a real red wine sample (Section 2.3).

### 2.1. PANI Affinity for 4-EP and 4-EG in Wine-Like Model Solution

**Figure 2:**
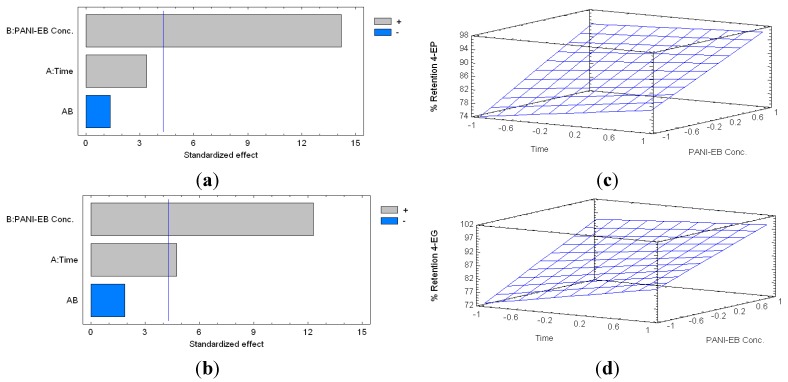
Standardized Pareto charts indicating the percentage of retention of 4-ethyphenol (4-EP) (**a**); and 4-ethylguaiacol (4-EG) (**b**) by PANI-EB treatment (Where: A, time of reaction; B, concentration of PANI-EB; and AB, interaction. The blue line represents the critical *t-*value, 95% confidence, with10 total degrees of freedom); and estimated response surfaces of 4-EP (**c**) and 4-EG (**d**) retention.

When model solutions containing 4-EP and 4-EG were treated with PANI-EB, the percentage of removal of 4-EP varied from 72.83% (8 h, 10 mg·mL^−1^) to 94.67% (24 h, 40 mg·mL^−1^), whilst the retention rate of 4-EG, ranged between 69.75% (2 h, 10 mg·mL^−1^) and 96.56% (24 h, 40 mg·mL^−1^). The Pareto charts obtained for this trial (Figure 2) show the statistical significance of the experimental variables (i.e., polymer concentration, B; contact time between the polymer and the sample, A; and their interaction, AB), and their respective estimated response surfaces. The vertical blue line of the Pareto charts signifies the limit of significance at 95% confidence. If the horizontal bars of the Pareto charts, representing the experimental variables (i.e., A, B, and AB) surpass the blue vertical line (i.e., limit of significance at 95% confidence, with 10 total degrees of freedom), it means that that variable is considered statistically significant and was included in the equation of the respective model. Also, different colors indicate whether the influence of these variables is positive or negative (i.e., whether they contribute or not to a higher retention of the volatile phenols), influences that are represented in the estimated response surface plots.

In the case of 4-EP, the concentration of PANI-EB is the only factor that showed a positive, and statistically significant influence over its retention. In the case of 4-EG, both factors (i.e., reaction time and PANI concentration) are statistically significant and have a positive influence on the retention rate. Therefore, the simplified equations of the models obtained were:

(1) Percentage of Retention 4−EP=86.3+8.99×CEB

(2) Percentage of Retention 4−EG=87.7+3.73×T+9.24×CEB

where *CEB* corresponds to PANI-EB concentration and *T* represents the contact time given to the polymer. The adjusted coefficients of determination (R^2^) of these models are 82.86% and 77.72%, respectively.

From these models, and based on our previous work [18], we chose a polymer contact time of 8 h for further trials. In the case of 4-EP, the contact time did not show to be significant. Therefore, the time chosen was based on the results of 4-EG (i.e., where high retention of the volatile phenols was observed), and considering a treatment that would be suitable for the laboratory work scale. Regarding the concentration of the polymer; in the case of 4-EP, 47.87 mg·mL^−1^ are required to obtain a significant retention level of the volatile phenols. In the case of 4-EG, 50.93 mg·mL^−1^ of PANI-EB would be required. Thus, when using a dose of 51 mg·mL^−1^ of PANI-EB, both compounds would be highly retained.

**Figure 3:**
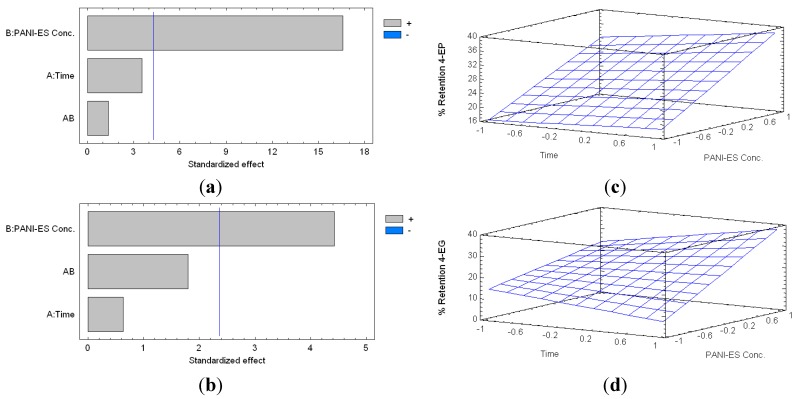
Standardized Pareto charts indicating the percentage of retention of 4-EP (**a**); and 4-EG (**b**) by PANI-ES treatment (Where: A, time of reaction; B, concentration of PANI-ES; and AB, interaction. The blue line represents the critical *t*-value, 95% confidence, with 10 total degrees of freedom); and estimated response surfaces of 4-EP (**c**) and 4-EG (**d**) retention.

With regards to PANI-ES, it was found that the percentage of retention of 4-EP ranged from 12.02% (2 h, 10 mg·mL^−1^) and 40.46% (24 h, 40 mg·mL^−1^). Moreover, the percentage of retention of 4-EG, varied between 0% (24 h, 10 mg·mL^−1^), and 36.95% (24 h, 40 mg·mL^−1^) (Figure 3).

Figure 3 shows that the retention of both compounds depends mainly on the concentration of PANI-ES. The simplified equations models in this case are:

(3) Percentage of Retention 4−EP=26.4+9.06×CES

(4) Percentage of Retention 4−EG=21.1+10.4×CES

where *CES* is PANI-ES concentration. The adjusted coefficients of determination (R^2^) of these models are 72.72% and 63.54%, respectively.

From these models, we can estimate the concentration of PANI-ES required to maximize the extraction of 4-EP and 4-EG, resulting in 146.754 mg·mL^−1^ of PANI-ES in the case of 4-EP, and 139.139 mg·mL^−1^ in the case of 4-EG. Therefore, a dose of 147 mg·mL^−1^ of PANI-ES should allow a significant removal of 4-EP and 4-EG under these experimental conditions. Since the time of interaction (polymer contact time) was not statistically significant, we decided to use the same time as in the previous experiment (8 h).

### 2.2. The Ability of PANI Polymers to Remove 4-EP and 4-EG from Wine-Like Model Solutions Containing Gallic Acid and 4-Methylcatechol

Here, the effect of having other phenolic compounds (i.e., Gallic acid, GA and 4-methylcatechol, 4MC) as part of the model wine, which potentially could be removed with the PANI polymers, was tested. When PANI-EB was used, the percentage of retention of 4-EP ranged between 39.98% (2 h, 10 mg·mL^−1^) and 94.83% (24 h, 40 mg·mL^−1^), whilst the retention rate of 4-EG ranged from 32.78% (2 h, 10 mg·mL^−1^) and 96.67% (24 h, 40 mg·mL^−1^). On the other hand, the retention percentage of GA varied between 85.04% (2 h, 10 mg·mL^−1^) and 99.89% (8 h, 40 mg·mL^−1^), whereas 4-MC ranged between 50.61% (2 h, 10 mg·mL^−1^) and 99.99% (24 h, 20 mg·mL^−1^).

**Figure 4:**
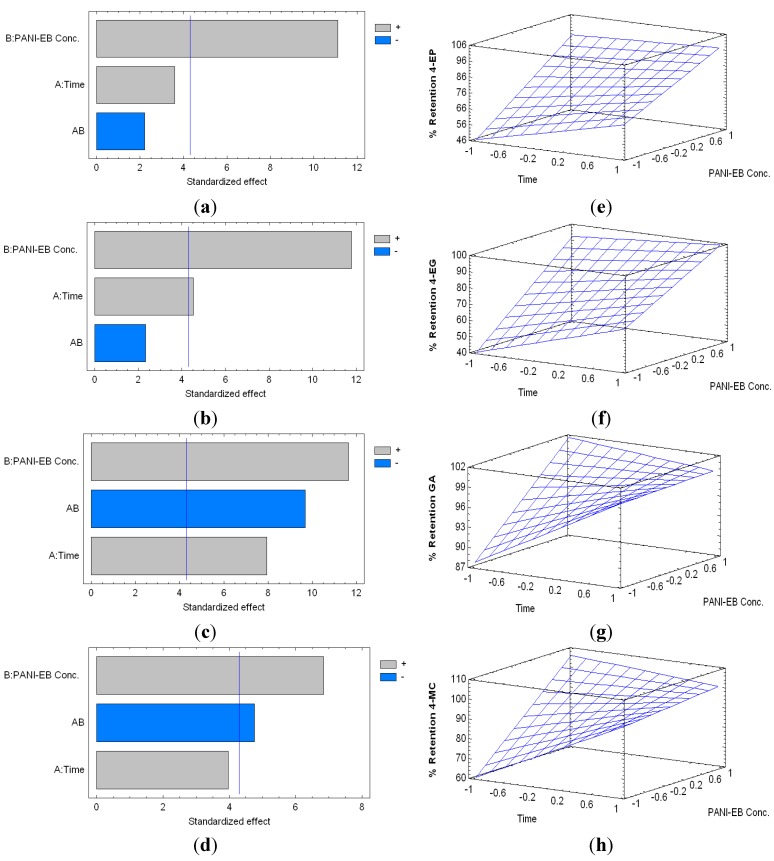
Standardized Pareto charts indicating the percentage of retention of 4-EP (**a**); 4-EG (**b**); gallic acid (GA) (**c**) and 4-methylcatechol (4-MC) (**d**); by PANI-EB treatment, respectively (Where: A, time of reaction; B, concentration of PANI-EB; and AB, interaction. The blue line represents the critical *t*-value, 95% confidence, with 10 total degrees of freedom); and estimated response surfaces of 4-EP (**e**); 4-EG (**f**); GA (**g**) and 4-MC (**h**) retention.

The standardized Pareto charts for the percentage of retention of 4-EP, 4-EG, GA and 4-MC are shown in Figure 4, along with their respective estimated response surfaces.

The percentages of retention of all the compounds studied were mainly influenced by the PANI-EB concentration, which exerted a positive influence on their removal. The factor “time” exerted a positive, and statistically significant influence, only in the case of 4-EG and GA. Based on these results, the simplified equations for the models are:

(5) Percentage of Retention 4−EP=76.4+19.5×CEB

(6) Percentage of Retention 4−EG=74.9+8.17× T +21.7×CEB

(7) Percentage of Retention GA=97.2+2.42×T+3.63×CEB−3.42×T×CEB

(8) Percentage of Retention 4−MC=90.9+12.9×CEB−10.3×T×CEB

where *T* is time of interaction, and *CEB* is PANI-EB concentration. The adjusted coefficients of determination (R^2^) of these models are 82.76%, 84.79%, 71.49%, and 59.46%, respectively.

From these models, considering a polymer contact time of 8 h, the predicted concentration of PANI-EB for the maximum removal of 4-EP and 4-EG are found to be 43.16 mg·mL^−1^ and 44.89 mg·mL^−1^ respectively.

**Figure 5:**
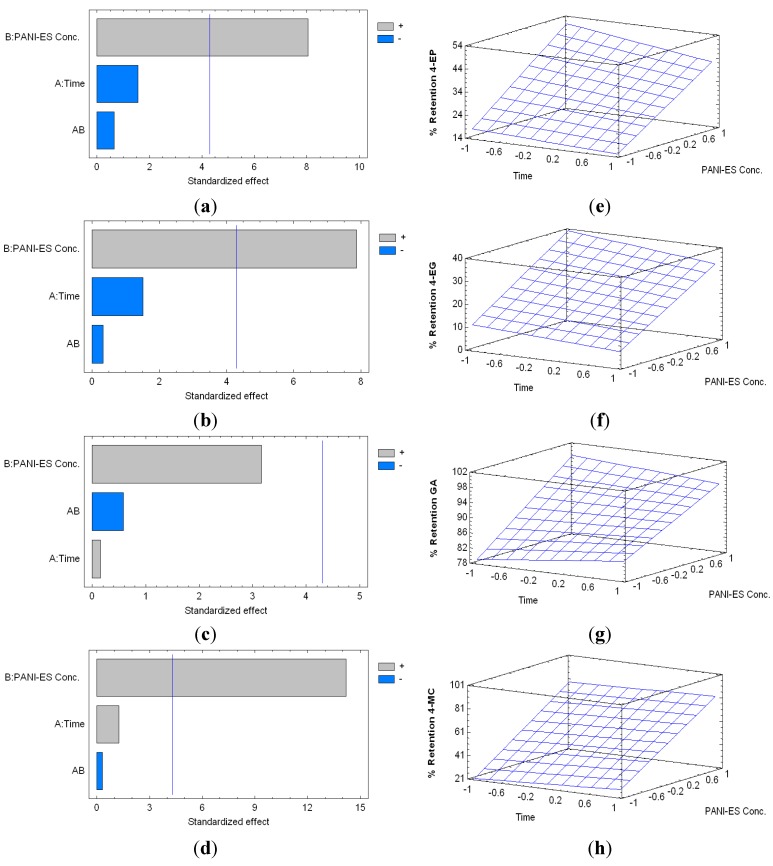
Standardized Pareto charts for Percentage of Retention of 4-EP (**a**); 4-EG (**b**); GA (**c**) and 4-MC (**d**), by PANI-ES treatment, respectively (Where: A, time of reaction; B, concentration of PANI-ES; and AB, interaction. The blue line represents the critical *t*-value, 95% confidence, with 10 total degrees of freedom); and estimated response surfaces of 4-EP (**e**); 4-EG (**f**); GA (**g**) and 4-MC (**h**) retention.

In the case of PANI-ES, the percentage of retention of 4-EP ranged between 13.53% (24 h, 10 mg·mL^−1^), and 50.45% (2 h, 40 mg·mL^−1^); between 6.73% (24 h, 10 mg·mL^−1^) and 39.02% (2 h, 40 mg·mL^−1^) for 4-EG; between 71.08% (2 h, 10 mg·mL^−1^) and 96.73% (2 h, 40 mg·mL^−1^) for GA; and between 16.16% (2 h, 10 mg·mL^−1^) and 81.75% (8 h, 40 mg·mL^−1^) for 4-MC (Figure 5). The PANI-ES concentration was statistically significant and has a positive influence (i.e., a higher retention) on the retention of 4-EP, 4-EG and 4-MC, whilst the polymer contact time was not statistically significant.

Based on these results, the simplified equations for the models are:

(9) Percentage of Retention 4−EP=31.7+15.3×CES

(10) Percentage of Retention 4−EG=22.7+13.7×CES

(11) Percentage of Retention GA=89.3

(12) Percentage of Retention 4−MC=52.6+28.4×CES

where *CES* is PANI-ES concentration. The adjusted coefficients of determination (R^2^) of these models are 94.02%, 93.61%, 62.12%, and 95.22%, respectively.

From these models, considering an interaction time of 8 h, the predicted concentration of PANI-ES to maximize the removal of 4-EP is 92.04 mg·mL^−1^. In the case of 4-EG, the predicted optimum PANI-ES concentration is 109.64 mg·mL^−1^. Considering 110 mg·mL^−1^ as PANI-ES dose, the model predicts the total removal of 4-MC from the model solution.

Therefore, both polymers tested were capable of removing volatile phenols, regardless of the presence of a high amount of non-volatile phenols in solutions (i.e., GA and 4-MC). The total concentration of non-volatile phenols (i.e., 2000 mg·L^−1^) was 20 times higher than that of volatile phenols, thus suggesting a higher degree of affinity of the polymers towards 4-EP and 4-EG.

### 2.3. The Affinity of PANI Polymers towards 4-EG and 4-EP in Red Wine

The wine sample selected had a total phenolic content of 2.03 ± 0.02 g·L^−1^ of GA equivalents and was spiked with 4-EP (5 mg·L^−1^) and 4-EG (5 mg·L^−1^). To be consistent with the previous experiments, this trial was conducted with 8 h of polymers contact time.

Table 1 show the concentration of 4-EP, 4-EG and total phenolics after a treatment with 0, 10, 25, 50 and 100 mg·mL^−1^ of PANI-EB and PANI-ES respectively. For each of the analytes of interest (i.e., 4-EP, 4-EG, and total phenolics), a single ANOVA test was performed including a simultaneous comparison of both polymers. The results of Table 1 indicate that PANI-EB is more effective in the removal of 4-EP and 4-EG, as previously observed from the affinity study. Moreover, it is observed that the total phenolic content decreases as the concentration of PANI increases. In the case of PANI-EB, this behavior is more pronounced than in the case of PANI-ES. This data is in good agreement with our previously published results, in which only 4-EG was evaluated [18].

**Table 1:** Theinfluence of the addition of PANI polymers on the removal of 4-EP, 4-EG, and total phenolics in a red wine sample.

PANI Dose [mg·mL^−1^]	4-EP Concentration [mg·L^−1^]		4-EG Concentration [mg·L^−1^]		Total Phenolics [g·L^−1^]	
	PANI-ES	PANI-EB	PANI-ES	PANI-EB	PANI-ES	PANI-EB
0	5.00 ± 0.06 i	5.00 ± 0.06 i	5.00 ± 0.04 g	5.00 ± 0.04 g	2.03 ± 0.02 f	2.03 ± 0.02 f
10	4.65 ± 0.03 h	1.61 ± 0.02 e	4.81 ± 0.12 g	2.55 ± 0.07 d	1.60 ± 0.06 e	1.19 ± 0.04 d
25	4.12 ± 0.02 g	0.86 ± 0.01 c	3.56 ± 0.01 f	1.00 ± 0.02 b	1.16 ± 0.05 d	0.29 ± 0.06 b
50	1.95 ± 0.04 f	0.320 ± 0.001 b	3.35 ± 0.01 e	0.37 ± 0.05 a	0.45 ± 0.03 c	0.22 ± 0.02 b
100	1.08 ± 0.01 d	0.000 ± 0.001 a	1.70 ± 0.15 c	0.29 ± 0.03 a	0.10 ± 0.02 a	0.0010 ± 0.0002 a

Results indicate the average (*n* = 3) ± standard deviation values. Statistical analyses were made separately for each compound. The same letters beside standard deviation denotes the absence of statistical differences using Tukey HSD, at 95% confidence level).

In the case of 4-EG, the treatments with PANI-ES were less effective in reducing its concentration than PANI-EB. In the case of total phenolic content, there were significant differences between all the PANI doses. For instance, at 10 mg·mL^−1^ of PANI-ES, the concentrations of total phenols are reduced 22.33% whilst PANI-EB removes 41.38%. Therefore, unless these polymers are used as fining agents aiming to significantly decrease the concentration of phenolic compounds in a wine sample, the treatments intended to reduce the concentration of volatile phenols should be limited to a low dose of PANI, as to avoid significant sensory changes, or a reduction in the aging capacity of the product, as a result of a major phenolic loss. For instance, 10 mg·mL^−1^ of PANI-EB reduces 67.8% of 4-EP, 50% of 4-EG and 41.38% of total phenols indicating that even lower doses the PANI-EB should be studied.

The interaction of PANI materials with these types of analytes (4-EP, 4-EG, GA and 4-MC) are based on not covalent bonding and have frequently been attributed to the combination of hydrogen, hydrophobic bonding and acid-base interactions. Phenolic compounds are acidic in nature and PANI materials have several imine and amine nitrogen in their backbone, which has a general basic character [18]. We hypothesize that the acid-base interaction between these molecules may be the predominant one. This could be justified by the superior removal capacity of PANI-EB over PANI-ES, because PANI-EB has more free available amine and imine nitrogens than PANI-ES. In case of PANI-ES, imine nitrogens have been already protonated with acid groups and are not available for further interactions with phenolic compounds.

## 3. Experimental Section

### 3.1. Chemical Reagents

4-Ethylphenol, 4-EP (≥98%), 4-ethylguaiacol, 4-EG (≥98%), gallic acid, GA (≥98%) and 4-methylcatechol (4-MC) (≥95%) were purchased from Sigma Aldrich (Saint Louis, MI, USA); whilst the Folin reagent (2N), sodium carbonate (≥99.5%), ethanol (≥99.5%), acetonitrile (≥98%, HPLC grade) and PVDF syringe filters (0.45 μm) were purchased from Merck (Darmstadt, Germany). Ultrapure water (18.2 MΩ·cm^−1^) was generated with an ELGA-Purelab water purification system. QuEChERS (Quick Easy Cheap Effective Rugged Safe) Extract Pouches (Extraction Salts: Sodium Citrate 1 g (99.9%), disodium citrate sesquihydrate 0.5 g (99%), magnesium sulfate (MgSO_4_) 4 g (≥98.5%), sodium chloride 1 g (≥99.5%) and dispersive solid-phase extraction (d-SPE) tubes, containing 150 mg primary secondary amine (PSA) sorbent and 900 mg MgSO_4_ (d-SPE PSA Tubes), were purchased from Agilent Technologies (Santa Clara, CA, USA). Polyaniline-emeraldine salt (PANI-ES) and polyaniline-emeraldine base (PANI-EB) were synthesized in house as briefly explained below.

### 3.2. Synthesis and Characterization of PANI Polymers

Polyaniline-emeraldine salt (PANI-ES) and polyaniline-emeraldine base (PANI-EB) (Figure 1) were prepared as previously reported [18,21], and the products were authenticated by infrared spectroscopy (Nicolet Nexus 470 FT-IR. Thermo Scientific, Waltham, MA, USA) followed by a comparison of vibrational bands that show them to be consistent with previously reported spectra for polyaniline materials [18,22].

### 3.3. The Affinity of PANI Polymers towards 4-EP and 4-EG in Model Solution

A preliminary study, using a model solution spiked with 4-EP and 4-EG, was conducted to determinate the retention capacity of PANI polymers towards these substances. A 500 mL of model solution was prepared by adjusting the pH of a 12% ethanol solution (*v*/*v*) to pH 3.3 using tartaric acid (ca. 5 g·L^−1^) and a 1 mM solution of sodium hydroxide [23]. A 250 mL sample of this model solution was spiked with 4-EP and 4-EG up to a final concentration of 5 mg·L^−1^. The later concentration of volatile phenols is higher than commonly found in real wines, particularly regarding 4-EG which concentration is normally much lower than that of 4-EP [7,9,10]. The concentration of volatile phenols chosen in these trials was used to test the trapping capacity of PANI polymers and whether they are more selective towards one or the other volatile phenol.

Later, 1 mL aliquots of the spiked model solutions were used a control (0 mg·mL^−1^ of PANI treatment), or treated with different amounts of polymers as to reach 10, 20, and 40 mg·mL^−1^ of PANI-polymers. After the addition of polymer, the samples were agitated at room temperature (ca. 20 °C) for 2, 8 and 24 h. in closed 12 mL amber vials, using a rock motion agitator, operating at 20 rpm. Then, the samples were filtered through PTFE syringe filter (0.45 μm), retaining the polymer-volatile phenol complex. The control samples use in this trial contained only 4-EP and 4-EG, received no PANI addition, and were treated the same way as the samples in which PANI polymers were added.

The remaining free concentration of 4-EP and 4-EG after filtration was determined by High performance liquid chromatography with diode array detector, HPLC-DAD, as further explained below. The differences observed between the concentration of volatile phenols in the control and PANI treated samples represent the retained amount of 4-EP and 4-EG.

### 3.4. The Ability of PANI Polymers to Remove 4-EP and 4-EG from Wine-Like Model Solutions Containing Gallic Acid and 4-Methylcatechol

In this trial, besides the presence of 4-EP and 4-EG, the model solution contained gallic acid (GA) and 4-methylcatechol (4-MC) as potentially competing substances to be removed by PANI polymers. These phenolic compounds were chosen due to their availability and the fact that they are commonly used to simulate the content of phenolics in model wines [24,25]. The model solution consisted of an acidified 12% ethanol solution (as described in Section 3.3) containing GA (1000 mg·L^−1^), 4-MC (1000 mg·L^−1^), 4-EP (5 mg·L^−1^) and 4-EG (5 mg·L^−1^). The control samples (0 mg·mL^−1^ of PANI) were treated the same way as those that receive PANI additions.

The amount of PANI materials used, the contact time required between the polymer and the model wine, and the rest of the conditions used in this trial were the same as in the optimization study (Section 3.3). The concentrations of 4-EP, 4-EG and non-volatiles phenols were determined by HPLC-DAD, as explained below. Once again, the differences observed between the control and PANI treated samples was used to calculate the retained amount of 4-EP and 4-EG.

### 3.5. The Affinity of PANI Polymers towards 4-EP, 4-EG and Total Phenolics in Red Wine

A 250 mL sample of a commercial red wine (Cabernet Sauvignon 2014), with undetected levels of volatile phenols was spiked with 4-EP and 4-EG (5 mg·L^−1^ each). Six mL aliquots of this spiked wine were treated with different amounts of PANI materials (i.e., 10, 25, 50 and 100 mg·mL^−1^ of PANI-ES or PANI-EB). Experiments were performed in triplicate to minimize manual error. The polymers were allowed to interact with the wine for 8 h in amber vials (as suggested by the results from the optimization study), with agitation, at room temperature (ca. 20 °C), followed by filtration with 0.45 μm PTFE syringe filters (as detailed in Section 3.3).

For the control treatment (0 mg·mL^−1^ of PANI), 3 aliquots of 6 mL of the wine spiked with volatile phenols received no PANI additions, but were agitated and filtered following the same protocol than the PANI treated samples. 

The doses of PANI materials chosen in this case, were based on the results of the models obtained from the trials detailed in Section 3.3 and Section 3.4. Given these results, we standardized the treatments at 50 or 100 mg·mL^−1^ of polymers. Moreover, considering the results obtained in a previously published paper [18], two treatments with lower polymer concentrations of 10 and 25 mg·mL^−1^ were also included.

The remaining concentration of 4-EP, 4-EG and total phenols after the PANI treatments were determined by HPLC-DAD and the Folin-Ciocalteu methods, as explained below.

### 3.6. Analyses of 4-EP, 4-EG and Phenolic Compounds in Model Solutions and Red Wine

#### 3.6.1. Extraction of 4-EP and 4-EG

The extraction of 4-EP and 4-EG, prior to the analyses by HPLC-DAD was performed using a modified QuEChERS methodology [26]. In brief, 2.5 mL of acetonitrile were added to 5.0 mL of wine and mixed for 1 min using a vortex. Then, 2 g of QuEChERS extraction salts were added to the sample, vortexed for 1 extra minute, and centrifuged for 10 min at 6000 rpm. Afterwards, the supernatant was transferred into a d-SPE PSA tube, vortexed for 1 min and centrifuged at 6000 rpm for 10 min. Finally, the liquid obtained after this process was analyzed by HPLC-DAD.

#### 3.6.2. Liquid Chromatography Analyses

An HPLC-DAD system (Agilent Technologies 1260 Infinity. Santa Clara, CA, USA) was used for the quantification of 4-EP, 4-EG, GA and 4-MC. Separations were achieved using a reverse-phased LiChrocart^®^ 250-4 RP-18 (250 mm × 4 mm ID × 5 μm) column (Merck, Darmstadt, Germany), under the following conditions: A mobile phase consisting of acetonitrile and acetate buffer (10 mmol·L^−1^, pH 4.7) operated in gradient mode at a flow rate was 0.7 mL·min^−1^. From 0 to 25 min, acetonitrile was increased linearly from 30 to 35%; from 25 to 30 min acetonitrile was increased to 50%. The initial chromatographic conditions were reached within 5 min and kept for additional 5 min before the next injection. The injection volume used was 20 μL, and analyte detection was done at 280 nm.

The quantification of 4-EP and 4-EG was done using a 10 point calibration curve ranging from 0.5 and 12 mg·L^−1^ of each volatile phenol. The resulting limits of detection (LOD) and limits of quantitation (LOQ) were as follows: For 4-EP, 0.024 and 0.080 mg·L^−1^ respectively; whilst for 4-EG, 0.008 and 0.025 mg·L^−1^ respectively (further details in supplementary information section).

#### 3.6.3. Total Phenolic Content

The measurement of total phenolics was achieved using the Folin-Ciocalteu method [27], measuring the absorbance of a Folin-reagent treated sample at 725 nm by means of a Spectroquant Pharo 300 UV–Visible spectrophotometer (Merck, Darmstadt, Germany). The concentration of total phenolics was estimated based on a standard curve of gallic acid (0–500 mg·L^−1^).

### 3.7. Experimental Design and Statistical Analyses

All the trials were designed as completely randomized experiments with treatments replicated thrice. The statistical evaluations consisted of performing an optimization of the variables involved (PANI dose and contact time). Thus, the variables were coded between −1 and 1, to give the same statistical weight. The results of the last experiment were analyzed by means of an analysis of variance, ANOVA, with mean separation performed by Tukey HSD test (95%). The software utilized was StatGraphics Centurion XV.

## 4. Conclusions

Both of the polymers tested (i.e., PANI-EB or PANI-ES) were capable of reacting and removing 4-EP and 4-EG from acidic model solutions and red wine, with PANI-EB being more effective than PANI-ES. The concentration of other phenolic species also declined as a result of the PANI treatments; however, in spite of their much higher concentration, they did not prevent the removal of volatile phenols (i.e., non-volatile phenols had a concentration 20 times higher than that of 4-EP and 4-EG combined). Therefore, the treatments intended to reduce the concentration of volatile phenols should be limited to a low dose of PANI, as to avoid significant sensory changes, or a reduction in the aging capacity of the product, as a result of phenolic losses.

Future studies should aim at evaluating other potential changes in the chemistry and sensory features of the wine treated, as well as developing other types of PANI materials.
