# Erratum: Carrasco-Sánchez, V.; et al. Removal of 4-Ethylphenol and 4-Ethylguaiacol with Polyaniline-Based Compounds in Wine-Like Model Solutions and Red Wine. *Molecules* 2015, *20*(8), 14312–14325

**DOI:** 10.3390/molecules22111890

**Published:** 2017-11-07

**Authors:** Verónica Carrasco-Sánchez, Amalraj John, Adolfo Marican, Leonardo S. Santos, V. Felipe Laurie

**Affiliations:** 1 School of Agricultural Sciences, Universidad de Talca, Talca 3460000, Chile; vecarrasco@utalca.cl; 2 Laboratory of Asymmetric Synthesis, Chemistry Institute of Natural Resources, Universidad de Talca, Talca 3460000, Chile; johnamalraj@gmail.com (A.J.); amarican@utalca.cl (A.M.); lssantos@utalca.cl (L.S.S.); 3 Nanobiotechnology Division at Universidad de Talca, Fraunhofer Chile Research Foundation—Center for Systems Biotechnology, FCR-CSB, Talca 3460000, Chile

The authors wish to make the following change to their paper [1]. The Acknowledgments section is incorrect in the published paper. The correct Acknowledgments should be as follows:

Postdoctoral Fondecyt Project 3140295, Proyecto Anillo (Integración de la Biología Estructural al desarrollo de la Bionanotecnología, ACT 1107), Innova Chile CORFO (Grant FCR-CSB 09CEII-6991), Programa de inserción y atracción CONICYT-79090038, and PIEI-QUIBIO (UTalca) are acknowledged. A.J acknowledges FONDECYT 11130087.

We apologize for any inconvenience caused to the readers by this mistake.
